# Use of Multiple Correspondence Analysis and K-means to Explore Associations Between Risk Factors and Likelihood of Colorectal Cancer: Cross-sectional Study

**DOI:** 10.2196/29056

**Published:** 2022-07-19

**Authors:** Dídac Florensa, Jordi Mateo-Fornés, Francesc Solsona, Teresa Pedrol Aige, Miquel Mesas Julió, Ramon Piñol, Pere Godoy

**Affiliations:** 1 Department of Computer Science University of Lleida Lleida Spain; 2 Department of Computer Systems Santa Maria University Hospital Lleida Spain; 3 Hospital-based Cancer Registry Arnau de Vilanova University Hospital Lleida Spain; 4 Catalan Health Service Department of Health Lleida Spain; 5 Biomedical Institute Research of Lleida Lleida Spain; 6 Centro de Investigación Biomédica en Red Madrid Spain; 7 Santa Maria University Hospital Population Cancer Registry Lleida Spain

**Keywords:** colorectal cancer, cancer registry, multiple correspondence analysis, k-means, risk factors

## Abstract

**Background:**

Previous works have shown that risk factors are associated with an increased likelihood of colorectal cancer.

**Objective:**

The purpose of this study was to detect these associations in the region of Lleida (Catalonia) by using multiple correspondence analysis (MCA) and k-means.

**Methods:**

This cross-sectional study was made up of 1083 colorectal cancer episodes between 2012 and 2015, extracted from the population-based cancer registry for the province of Lleida (Spain), the Primary Care Centers database, and the Catalan Health Service Register. The data set included risk factors such as smoking and BMI as well as sociodemographic information and tumor details. The relations between the risk factors and patient characteristics were identified using MCA and k-means.

**Results:**

The combination of these techniques helps to detect clusters of patients with similar risk factors. Risk of death is associated with being elderly and obesity or being overweight. Stage III cancer is associated with people aged ≥65 years and rural/semiurban populations, while younger people were associated with stage 0.

**Conclusions:**

MCA and k-means were significantly useful for detecting associations between risk factors and patient characteristics. These techniques have proven to be effective tools for analyzing the incidence of some factors in colorectal cancer. The outcomes obtained help corroborate suspected trends and stimulate the use of these techniques for finding the association of risk factors with the incidence of other cancers.

## Introduction

Colorectal cancer is the third most common type of cancer worldwide [1,2]. In Europe, around 250,000 new colorectal cancer cases are diagnosed each year, accounting for around 9% of all malignancies. The rates of this cancer increase with industrialization and urbanization. In general, the evidence shows that the incidence increases in countries where the overall risk of large bowel cancer is low, while in countries with high incidence, the rate has either stabilized or decreased, particularly among younger age groups [3].

In the province of Lleida (Spain), the population-based cancer registries allow the identification and counting of all incident cases (new cases) diagnosed among the residents of this geographical area [4]. The residents of the Lleida region present lifestyles, risk factors, and work activity, which can be used to determine the specific incidence of certain types of cancer. Nearly half the population of the Lleida province live in rural and semiurban areas. As a consequence, their lifestyle is different from that of the more urban populations in other Catalan provinces [5,6]. Thus, they can present different risk factors and socioeconomic status (SES).

Some studies have demonstrated a higher incidence of colorectal cancer among those with low SES and risk factors such as BMI and smoking. A pooled European cohort study [7] demonstrated that adult weight gain was associated with increased risk of several major cancers. They also concluded that the degree, timing, and duration of being overweight and obesity also seemed to be important. More specifically for colon cancer, Guo et al [8] presented a prospective cohort study in northern China. They concluded that obesity increased the risk of colon cancer in males. Regarding smoking, Mizoue et al [9] presented a report evaluating the association in the Japanese population based on a systematic review of epidemiological evidence. This report concluded that tobacco smoking may increase the risk of colorectal cancer in the Japanese population. However, there is still insufficient epidemiological evidence to demonstrate any clear association with colon cancer. Kim et al [10] studied a possible association between SES and the risk of colorectal cancer in women. Their findings suggested that high SES may protect against colorectal cancer in women. The methodology used in these studies was similar, namely, the multivariate regression analysis.

Recent research has applied the techniques used in this study, but none of these studies were for cancer and risk factors. Ugurlu and Cicek [11] used the multiple correspondence analysis (MCA) method to search for relations in ship collisions [11]. However, the k-means algorithm was more widely used in some cancer aspects. Rustam et al [12] applied this technique to obtain the centroid of each cluster and predict the class of every data point in the validation set. Recently, Ronen et al [13] used k-means as an initial step in a deep learning method to evaluate the colorectal cancer subtypes. K-means allowed the detection of relevant clinical patterns that improved the prediction model. Therefore, the use of MCA and k-means to search for the relationship between risk factors and cancer incidence is a novel method.

Several studies [7-10] have found new associations among risk factors, demographic information, and SES in patients with colorectal cancer. These studies have taken a great effort to analyze and compare risk factors such as obesity, cigarette smoking, and SES in patients with colorectal cancer. They used statistical methods, including Cox regression, Spearman rank correlation coefficient, and multilevel logistic regression to estimate the association between variables. However, none of them used a combination of a statistical method like MCA and an artificial intelligence algorithm such as k-means to search for associations between a group of categorical variables.

As the main contribution of this study, we propose the use of MCA as a statistical technique to detect relations between risk factors and patients’ characteristics and k-means as an unsupervised learning algorithm to search for clusters of patients with similar risk factor profiles for colorectal cancer.

## Methods

### Preprocessing

**Figure 1:**
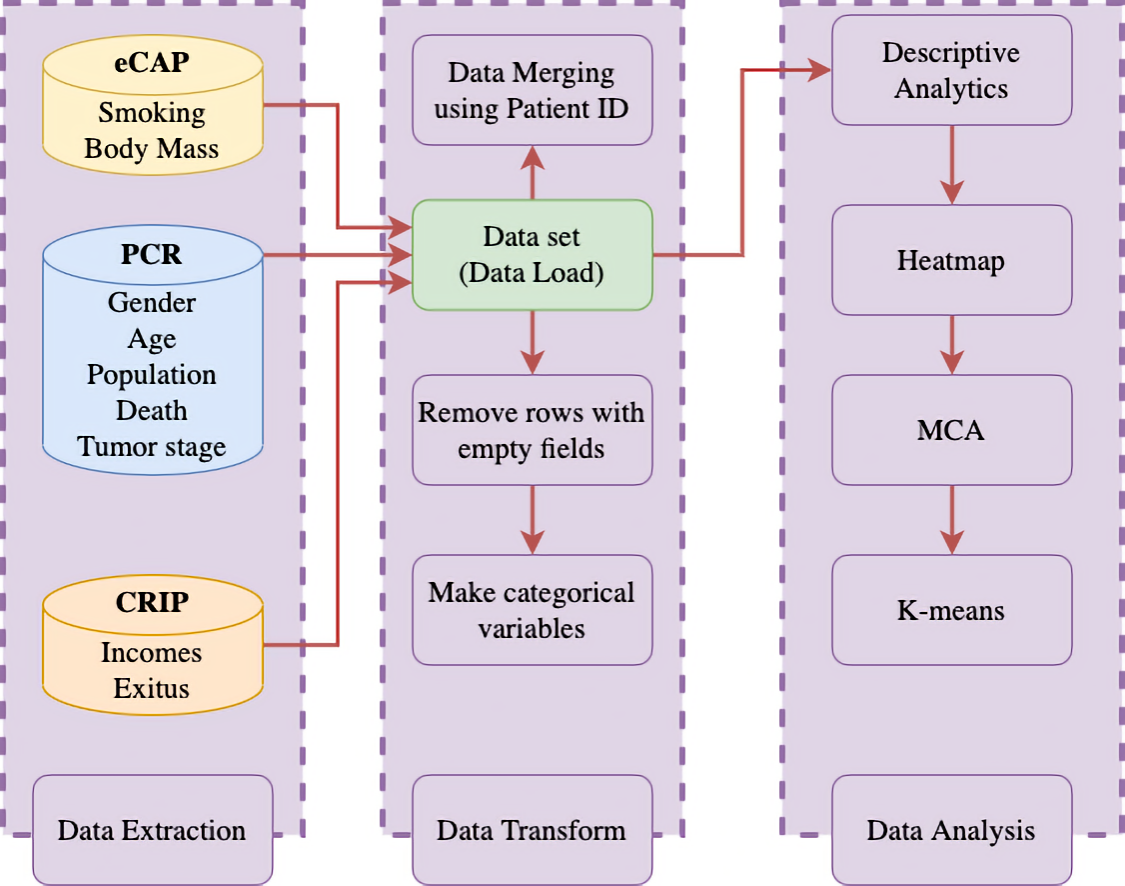
System flow chart. CRIP: Central Register of Insured Persons; PCR: population-based cancer registry.

The main information sources were the population-based cancer registry of the health region of the province of Lleida, the eCAP (a computerized medical history program used by doctors, pediatricians, and nurses in primary care centers when they see their patients [14]) software, and the Central Register of Insured Persons (a register that allows the unique identification of those covered by the Catalan Health Service through the personal identification code, the management and consultation of their data, and their updates [15]). Before applying the statistical technique, the information was validated by experienced professionals (doctors, nurses, and documentalists) in the Lleida population-based cancer registry who reviewed the clinical history of each patient. After that, the International Agency for Research on Cancer tool was applied to detect unlikely or impossible codes or combinations of codes [16]. Then, an accurate description of the data and basic concepts of the MCA and k-means used in this work are explained in this section. See the system flow chart of the whole process in Figure 1; it shows the different registers used to extract the data, its process and transformation, and its applied analysis. The patients with empty fields were removed.

### Study Population

The colorectal cancer data were extracted from the new cases registered between 2012 and 2015 in the Lleida population-based cancer registry [5,17,18] for patients with cancer in the main hospitals in the health care region of the Lleida province. Specifically, the data set consisted of 1083 new colorectal cancer cases. These hospitals were the Arnau de Vilanova University Hospital and the Santa Maria University Hospital, and the primary information sources were hospital records (International Classification of Diseases, ninth revision codes-140.0 to 208.9) and reports from pathological anatomy. Additionally, these reports confirmed >92% of cases included in the sample. Risk factors such as BMI and smoking were extracted from eCAP software and the SES was extracted from the Central Register of Insured Persons. The study is compliant with the General Data Protection Regulation (European Union), thereby maintaining the anonymity of the patients. Cancer episodes were recorded according to international criteria. In addition, the data analysis (done with R) can be freely downloaded from this GitHub repository [19]. It also included a mock data set randomly generated to test the models. The original data set could not be uploaded due to General Data Protection Regulation, which does not permit sharing patients’ information.

The BMI was used to calculate the obesity of each patient by standard weight status categories [20]. We categorized the BMI as the established table: <24.9 as normal weight, 25-29.9 as overweight, and >30 as obesity. Regarding SES, we categorized the variable according to the annual income available from the Central Register of Insured Persons. According to the legislation [21], we created 2 groups: annual income <€18,000 (low income) and >€18,000 (high income) (€1=US $1.04). The population was categorized as rural, semiurban, and urban. In accordance with [22], people living in cities with a population of more than 10,000 were classified as urban, population between 10,000 and 2000 in towns as semiurban, and the rest as rural. The Spanish National Statistics Institute has defined rural areas as those with a population of less than 2000, semiurban areas as those with a population between 2001 and 10,000, and urban areas as those with a population with more than 10,000 people. All the cancer cases that did not conform to one of these fields were discarded automatically. To sum up, each register contains the following fields: age group (50-64 years, 65-74 years, ≥75 years); gender (male, female); population (rural, semiurban, urban); exitus (death, alive); BMI (normal, overweight, obesity); smoking (ex-smoker/smoker, nonsmoker); income (high income, low income); and stage (0, I, II, III, undefined). Table 1 shows the number of cases for each category.

**Table 1:** Principal comorbidities groups included in this study: patients with colorectal cancer between 2012 and 2015, where all the comorbidities were properly registered (N=1083).

Characteristics			Values, n (%)
**Gender**			
	Male	689 (63.6)	
	Female	394 (36.4)	
**Age group (years)**			
	50-64	319 (29.5)	
	65-74	328 (30.3)	
	≥75	436 (40.2)	
**Exitus**			
	Death	221 (20.4)	
	Alive	862 (79.6)	
**Income^a^**			
	<€18,000/year	863 (79.7)	
	>€18,000/year	220 (20.3)	
**Population**			
	Rural	228 (21.1)	
	Semiurban	333 (30.7)	
	Urban	522 (48.2)	
**BMI**			
	Normal	234 (21.6)	
	Overweight	506 (46.7)	
	Obesity	343 (31.7)	
**Smoker**			
	Smoker/Ex-smoker	232 (21.4)	
	Nonsmoker	851 (78.6)	
**Stage**			
	0	64 (5.9)	
	I	115 (10.6)	
	II	168 (15.5)	
	III	91 (8.4)	
	Undefined	645 (59.6)	

^a^€1=US $1.04.

### MCA Algorithm

MCA is an unsupervised learning algorithm for visualizing the patterns in large and multidimensional categorical data [23]. This method can be used to analyze, explore, summarize, and visualize information contained of individuals described by categorical variables [24]. Unlike correspondence analysis (CA), MCA can deal with more than one categorical variable. This is the main advantage of the MCA technique. In our case, MCA was first used to evaluate the relationships between all the features. MCA was then used to evaluate the relationships among population, age, gender, exitus, BMI, smoking, and tumor stage. Associations between features are represented graphically [25]. The graphs aim to visualize the similarities or differences in the profiles simultaneously, identifying those dimensions that contain most of the data variability. Features or their categories close to each other are significantly related statistically.

The factors were interpreted with the help of various statistical coefficients, which complemented each other to provide a better interpretation. The most common and important are inertia, eigenvalue, contribution, and factorial coordinates. Inertia is a measurement of the dispersion of the set of computed distances between points. Analogously, in principal CA, inertia corresponds to the explained variance of dimensions. The eigenvalue allows the inertia that a specific category produces to be quantified determining a certain percentage relative to the entire set of the active category. The percentage coordinates (x- and y-axis) of the graph enable the category points in a graph to be represented and established. In MCA, the distance between 2 or more categories of different variables can be interpreted in terms of the associations and correlations between these. If 2 categories present high coordinates and are close in space, this means that they tend to be directly associated [26,27]. If 2 categories present high coordinates but are distant from each other (eg, they have opposite signs), this means that they tend to be inversely associated [28,29]. A heatmap was created to help the interpretation of the MCA. This plot used the intensity of the colors to show the level of association between the variables. Our graphs showed the association by the distance between the categories in the MCA plot.

### K-means

K-means [30] is a nonsupervised learning algorithm used in data mining and pattern recognition. The algorithm partitions the data set in *k* predefined distinct nonoverlapping subgroups (clusters) where each data point belongs to only one group. It tries to make the intracluster data points as similar as possible while also keeping the clusters as different (far) as possible. It assigns data points to a cluster such that the sum of the squared distance between the data points and the cluster’s centroid is at the minimum. The less variation we have within clusters, the more homogeneity (similarity) there is between the data points within the same cluster. The k-means algorithm is composed of the following steps: (1) it places *k* points in the space represented by the patients who are being clustered, (2) it assigns each patient to the group that has the closest centroid, and (3) when all patients have been assigned, it recalculates the positions of the *k* centroids. Steps 2 and 3 are repeated until the centroids no longer move. This produces a separation of the patients into homogenous groups while maximizing heterogeneity across groups. The optimal number of clusters was obtained by the elbow method [31]. This consists of plotting the explained variation as a function of the number of clusters and picking the elbow of the curve as the number of groups to use. To assess internal cluster quality, cluster stability of the optimal solution was computed using Jaccard bootstrap values with 10,000 runs [32].

### Statistical Analysis

All the information presented was analyzed using MCA, an extension of CA, and the k-means algorithm. The combination of MCA and k-means benefits the effectiveness of the calculation process and, in consequence, the k-means results. MCA helps to reduce the noise, which allows the k-means algorithm to obtain more accurate distances. The MCA dimension reduction automatically performs data clustering according to the k-means objective function [33]. In addition, the potential confounding factors in this study were assessed by calculating the distances between the variables (inertia) that take into account their relative weight in the database as a whole. However, these variables were related to each other depending on the similarity of each register. Previously, the patients with empty fields were removed.

The MCA method was implemented in scripts performed with R [34], an open-source programming language and environment for statistical computing and graphics. Specifically, the main library used to implement the methods and obtain the results was FactoMineR [35]. K-means was written in Python [36], and the main library used scikit-learn [37]. These methods were launched by their default configuration and using a personal computer.

## Results

### MCA and K-means Without the Tumor Staging

**Figure 2:**
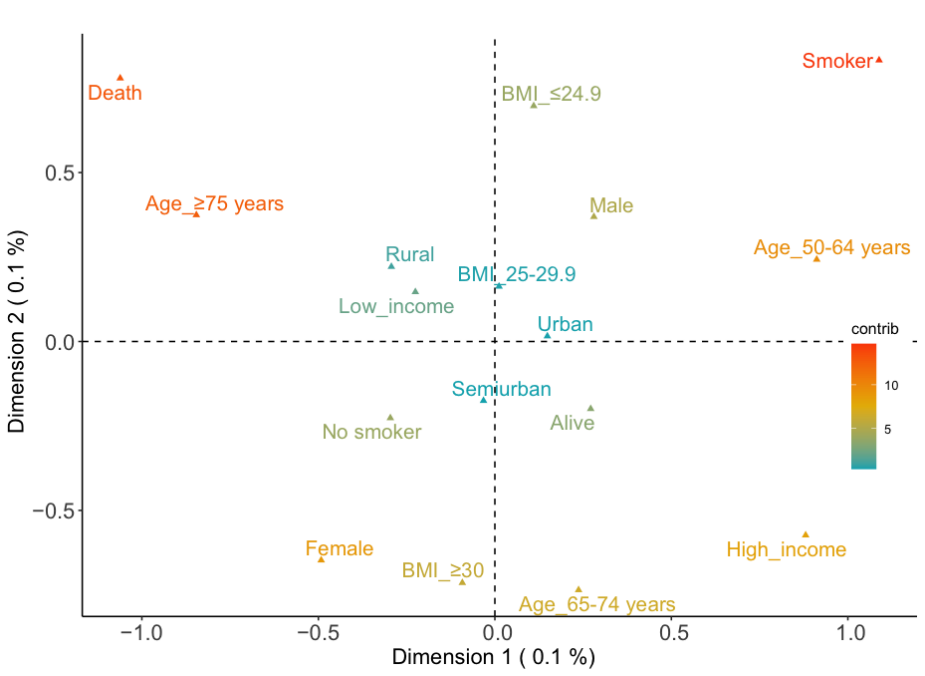
2D multiple correspondence analysis plot showing the correlations between the categories and their contributions for all data sets.

**Figure 3:**
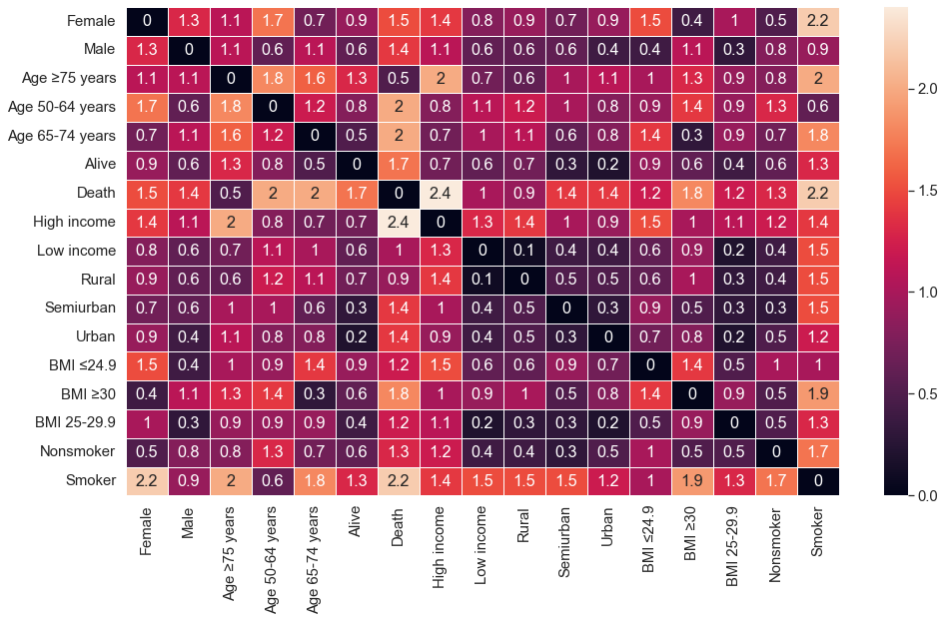
Correlations between the categories by the distance between them.

The analysis of the MCA and k-means without the stage variable included 1083 registers. Figure 2 shows the different categories and their possible associations. The variance for dimension 1 was 15% (eigenvalue 0.21) and that for dimension 2 was 12% (eigenvalue 0.17). Figure 2 also shows the position of each category in the plot and its contribution on the dimensions. Note the contribution of mortality (15% on the negative x-axis and 10.2% on the positive y-axis), the ≥75 years age group (18.8% on the negative x-axis and 4.5% on the positive y-axis), and the ex-smoker/smoker (16.5% and 12.3% on positive x-y axis). Figure 3 shows the relation between the categories. The associations between the points were significant when they were closer and the distance was minimum. For example, females and obesity were represented in the same dimension in the MCA plot. Therefore, the heatmap also demonstrated this association with a distance of 0.4 between the points in the MCA plot.

Graphically, the points closer to each other or the points represented in the same direction of the axis suggest associations. As can be seen, mortality and older age are very close in the plot. This suggests a possible association. Another possible relation observed could be between females and obesity. Then, a cloud on the positive x-axis and the negative y-axis was made up of the 65-74 years age group, high income, and survival. Finally, additional associations could be made up of the 50-64 years age group, males, smokers or ex-smokers, and normal weight.

Table 2 shows the centroids of the main clusters obtained after applying the k-means algorithm. The recommended number of optimal clusters was 5 [31] (see the GitHub [19] repository to evaluate the plot). The first cluster grouped 242 registers among which the main register was males aged ≥75 years from urban populations, with low income, nonsmokers who were overweight, and with a low risk of dying. The next cluster (259 registers) represented females aged between 50 and 64 years with high income. It grouped the cases from rural populations with normal weight and survival. Cluster number 3 was made up of 180 registers. These were mostly males aged ≥75 years with low income and from semiurban populations. They were nonsmokers but were obese and unfortunately included exitus. It was the only cluster that included mortality. The fourth cluster represented urban males aged between 65 and 74 years and with low income. In this case, they were smokers or ex-smokers with normal weight and no mortality. It contained 194 registers. Finally, the last cluster was made up of 208 cases, which included semiurban females aged between 65 and 74 years with low income. They were not smokers but they were overweight. Fortunately, surviving patients predominated in this cluster and the risk of dying was low. See these clusters represented graphically in the GitHub [19].

**Table 2:** Centroids of the main clusters obtained from the k-means algorithm for all data sets.

Cluster 1	Cluster 2	Cluster 3	Cluster 4	Cluster 5
Urban	Rural	Semiurban	Urban	Semiurban
Age ≥75 years	Age 50-64 years	Age ≥75 years	Age 65-74 years	Age 65-74 years
Low income	High income	Low income	Low income	Low income
Male	Female	Male	Male	Female
Nonsmoker	Nonsmoker	Nonsmoker	Smoker/Ex-smoker	Nonsmoker
Overweight	Normal weight	Obesity	Normal weight	Overweight
Alive	Alive	Death	Alive	Alive

### MCA and K-means Including the Tumor Staging

**Figure 4:**
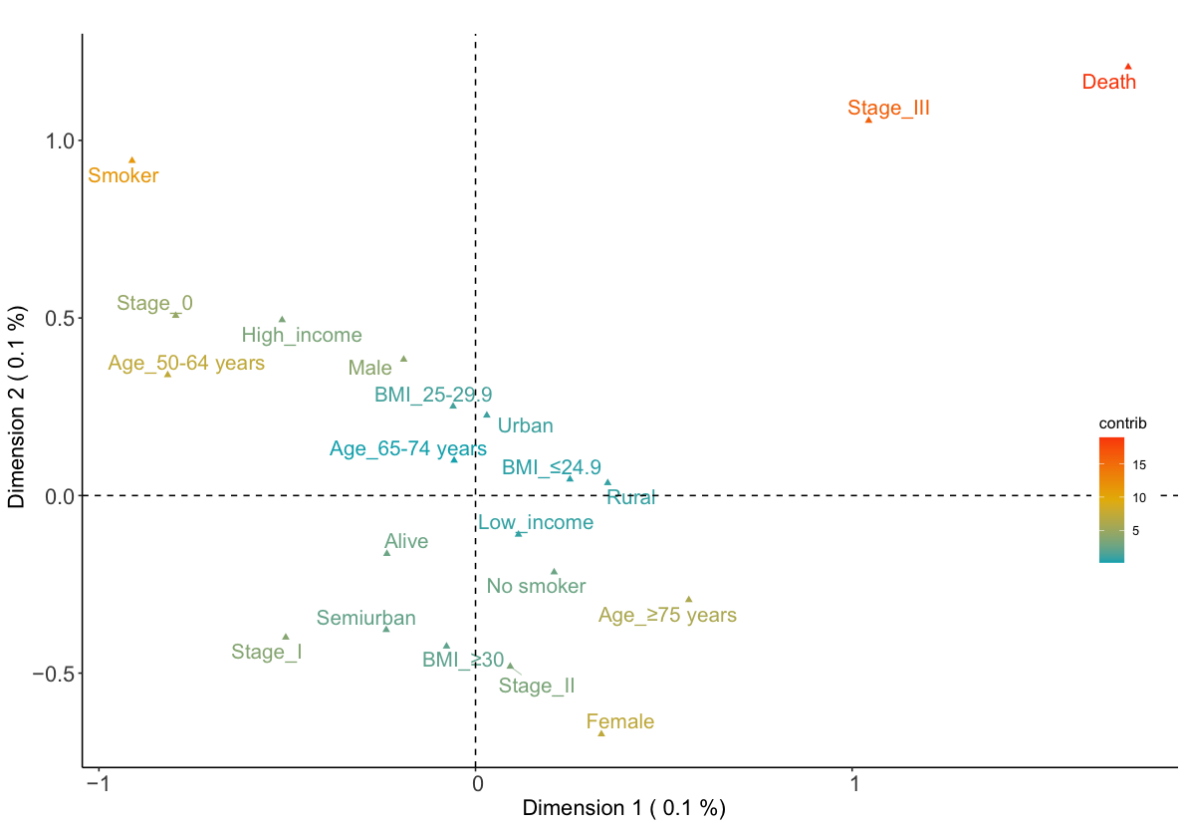
2D multiple correspondence analysis plot showing the correlations between the categories and their contributions.

**Figure 5:**
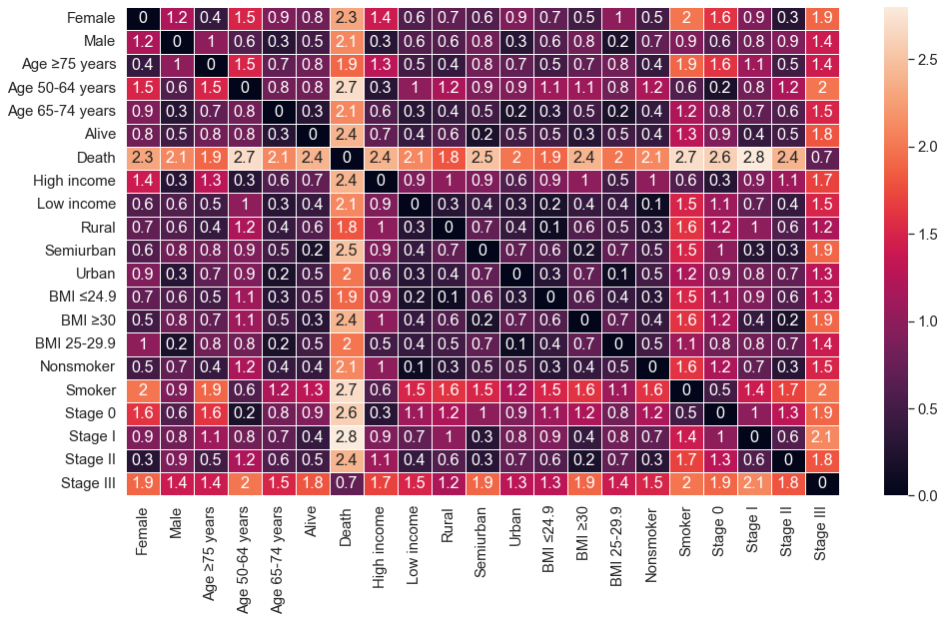
Correlations between the categories by the distance between them including the tumor staging.

This subsection presents the outcomes, including the stage of the tumor. The data set used for this analysis discarded the registers, which did not contain the stage (647 registers). Therefore, the number of cases analyzed was 438 (Table 1). Figure 4 shows the outcomes obtained after applying MCA. The variance of dimension 1 was 11.4% (eigenvalue 0.18) and that of dimension 2 was 10.2% (eigenvalue 0.16). Mortality was also one of those with the highest contribution (26.4% on the positive x-axis and 10.5% on the positive y-axis). Near this was stage III with a high contribution (16.3% on the positive x-axis and 13.7% on the positive y-axis). Ex-smoker/smoker contributed significantly compared with the rest of categories (9.1% on the negative x-axis and 1.3% on the positive y-axis). The relations between these and other categories are shown in Figure 5. See the death and its correlation between stage III. The heatmap differentiated this association clearly, as the MCA plot also showed. The location of the categories in the plot and their contributions suggested possible associations. The main association was between stage III and mortality and with females with stage II, the ≥75 years age group, and nonsmokers. Another relation could be males with high income, aged between 50 and 64 years, stage 0, and ex-smokers or smokers. However, these results could be affected by the decrease in cases.

Table 3 shows the clusters obtained from the data set with the tumor stage. All the clusters obtained were male nonsmokers owing to the decrease in the number of registers in the data set. The first cluster with 135 cases represented obese urban patients aged between 65 and 74 years with stage II reached and a low risk of death. The second cluster had 120 registers of patients with stage II and age ≥75 years from semiurban populations. Their risk of death was also low. The next cluster included 76 registers and they were from the urban population but overweight. They included the younger patients (50-64 years age group), with a low risk of death and the lowest stage (stage 0). The fourth cluster (n=72) represented rural inhabitants, aged between 65 and 74 years. They were obese with stage III cancer but low risk of death. However, the fifth cluster was patients from the semiurban population, aged ≥75 years, overweight, in an advanced stage (III), and with a high risk of death. See these clusters represented graphically in the k-means folder of GitHub [19].

**Table 3:** Centroids of the main clusters obtained from the k-means algorithm: the final data set after including the stage of the tumor.

Cluster 1	Cluster 2	Cluster 3	Cluster 4	Cluster 5
Urban	Semiurban	Urban	Rural	Semiurban
Age 65-74 years	Age ≥75 years	Age 50-64 years	Age 65-74 years	Age ≥75 years
High income	Low income	Low income	Low income	Low income
Male	Male	Male	Male	Male
Nonsmoker	Nonsmoker	Nonsmoker	Nonsmoker	Nonsmoker
Obesity	Obesity	Overweight	Obesity	Overweight
Alive	Alive	Alive	Alive	Death
Stage II	Stage II	Stage 0	Stage III	Stage III

## Discussion

The MCA technique and the k-means algorithm permit the analysis and detection of clusters of patients with similar risk factors and outcomes not observed in the literature. The population-based cancer registry for the province of Lleida registered 1083 colorectal cancers between 2012 and 2015. This cancer is the most incident in our region [5,17,18] and by applying MCA and k-means, some relationships were found between some aspects that corroborate the usefulness of these techniques. They helped to detect that in colorectal cancer, the age group and BMI risk factors are related. Another important corroboration was the risk of death in older people (≥75 years age group) either obese or overweight and in an advanced stage. Related to this latter factor, the advanced stage was observed in older people with obesity. Stages II and III were 65% (119/181) of the total in the ≥75 years age group.

Previous studies have used clustering techniques to detect associations, but none of them were used for associating patient profiles with risk factors. We based our study on a preliminary paper [38], which evaluated the relationship between air pollution, particulate matter components, and risk of breast cancer in a United States–wide prospective cohort by using a clustering technique. That study concluded that air pollution measures were related to both invasive breast cancer and ductal carcinoma in situ within certain geographic regions. Another starting point was the study presented in [39], which used the combination of MCA and k-means to ascertain multimorbidity patterns. That study concluded that these techniques could help to identify these patterns. Another study our work was based on is the one presented in [40], which studied the trends in the incidence of cancers associated with being overweight and obese. Another study [41] analyzed the possible relation between obesity and colorectal cancer. These papers studied the impact of the risk factors on colorectal cancer but did not use the MCA technique or k-means algorithm to explore associations between these and their impact. In addition, a previous study used MCA to analyze the prognosis in surgery for low rectal cancer [42]. Another study used k-means to search patterns in patients with colorectal cancer, but its main aim was to detect emotion regulation patterns and personal resilience [43]. However, to the best of our knowledge, no prior studies have used MCA or k-means to link types of risk factors, SES, tumor stage, and patients’ characteristics in cases of colorectal cancer.

One MCA outcome was the inertia (27%). Further, various variables had high contributions. A strong relation was obtained between older patients (≥75 years age group) and mortality. This may suggest an increase in the risk of mortality for colorectal cancer in older adults, as previous studies showed [44]. On the opposite side of previous associations, it showed another association between survival, high SES, and the 65-75 years age group. Even though the contributions of these are lower than those of mortality and the older population, it is suggested that the risk of death is lower in people with high SES [45] and among younger people. An association was detected between females and obesity although this was not reflected in the k-means. This relation may be because 37% (146/394) of all the women were obese. However, obese men represented 29% (205/689) of the male population, and the percentage of obesity in the data set was 31% (343/1083). This relation suggests that obese women could more likely develop colorectal cancer than men. In general, the probability of colorectal cancer in obese patients can increase by 30%-70% [46]. However, although the contribution is too low to establish a strong relation, the position of males and normal weight in the plot might suggest that there may be some other factors that increase the risk of this cancer and that these techniques highlighted other associations. Some additional patient clinical history would be necessary.

Regarding the k-means analysis, the third cluster confirmed the mortality in the older population with obesity [44]. The first cluster also represented the ≥75 years age group but who were overweight and had no exitus. These differences between clusters suggested that obesity may be a determining factor in older persons that increases the risk of death. In addition, these 2 clusters were males. Similar outcomes were obtained in the fifth cluster when the tumor stage was added. Stage III was directly related with the ≥75 years age group, the semiurban population, and mortality, thereby suggesting that for older persons, being overweight or obese and in an advanced stage could increase the risk of death. The fourth cluster was made up of smokers or ex-smokers. Although tobacco is not usually directly related with colorectal cancer, some studies also support this result [47,48].

The analysis then studied the data set filtered by tumor stage. The final data set was made up of 438 registers. The MCA technique obtained a significant relation between stage III and mortality. However, screening programs and technology decrease this risk, as recent studies concluded [49]. We can also see that stage 0 was related with younger people (50-64 years age group). The k-means results gave similar conclusions as in the MCA. The younger people, stage 0, and survival appeared in the same cluster as demonstrated in the previous k-means analysis with the second cluster. This suggests the importance of screening programs to detect tumors at an early stage [50]. The fourth cluster in the second analysis related rural and stage III. This association may insinuate a possible delay in diagnosis or difficulties in accessing the health care system and mass screening testing in rural areas [51]. Finally, note that all clusters that had stage II or III also included obesity or excess weight. This may suggest that the BMI may be a determinant for having an aggressive colorectal tumor. However, no significant outcomes related to income were obtained, although 80% (863/1083) of the cases were low-income patients. This high percentage of low-income cases could be explained by the fact that the average annual net income per person in Catalonia in 2015 was €12,283 [52].

The strengths of using the MCA and k-means cluster analysis are that the results are less susceptible to outliers in the data, the influence of chosen distance measures, or the inclusion of inappropriate or irrelevant variables [53]. This study had some limitations that should be noted. Regarding the techniques, it tends to take into account the relative weight of each variable concerning the set of study variables and allows control for potential confounding factors such as sex, age, and survival. However, some residual confounding effects cannot be ruled out. Further, these include the low number of cases with tumor stage (438/1083, 40% of total). In consequence, the final data set also made it difficult to analyze the strength of the causal relationship between different prediction parameters and outcomes because it contained few registers. The postal address registered for each case was the patient’s home address at the time of cancer diagnosis. However, this address may have changed during the study. Despite this, the number of cases with changed addresses would be very low and this factor is not expected to produce bias in the results. Some lifestyle aspects such as alcohol consumption, diabetes, or profession were not considered. The lack of cause of death is another limitation. The results showed that there is room for other kinds of risk factors. Additional patient clinical history would be required in order to find these. Further, related to the comorbidities, the Charlson index could not be added because approximately only 15% of the sample received it. A future study may be the study of the causality, adding synthetic data to enlarge the data set. Finally, some associations could hide others due to these techniques even though they showed the most significant relationships. In addition, the genetic and hereditary conditions were not considered.

In conclusion, many studies demonstrate that some risk factors such as BMI, tobacco smoking, or SES could influence the incidence of colorectal cancer by using traditional techniques. This study used new techniques such as MCA and k-means to analyze the relationships between colorectal cancer and risk factors. The outcomes obtained demonstrated that the combination of these techniques could help to detect relations between risk factors and patient characteristics. Obesity and being overweight in the older population (≥75 years age group) increases the risk of developing aggressive tumors and death. Stage 0 was related with younger people and survival. This highlights the importance of screening programs for colorectal cancer. The presence of tobacco in a cluster indicated that it must be considered as a risk factor in colorectal cancer. The results of our study help to corroborate suspected trends in several of the relationships detected and confirm the usefulness of these techniques. Further, they encourage applying these methods to other cancers and detecting how the risk factors could be associated. In future work, it is important to delve deeper into the patients’ characteristics and risk factors. This means including new variables such as diabetes, alcoholism, or the cause of death. The findings obtained in this study motivate us to search for relations between risk factors in other cancers. Moreover, new techniques and artificial intelligence algorithms can be implemented to explore patterns of pretumor and posttumor detection from the clinical history.

## Abbreviations

CA: correspondence analysis
MCA: multiple correspondence analysis
SES: socioeconomic status
